# Ectopic Blood Supply of Hepatocellular Carcinoma as Depicted by Angiography with Computed Tomography: Associations with Morphological Features and Therapeutic History

**DOI:** 10.1371/journal.pone.0071942

**Published:** 2013-08-15

**Authors:** Guang-wen Chen, Bin Song, Zhen-lin Li, Yuan Yuan

**Affiliations:** 1 Department of Radiology, Sichuan Provincial People’s Hospital, Chengdu, Sichuan, China; 2 Department of Radiology, West China Hospital of Sichuan University, Chengdu, Sichuan, China; Wayne State University, United States of America

## Abstract

**Objective:**

To investigate the associations of ectopic blood supply of hepatocellular carcinoma (HCC) with its morphological features and therapeutic history.

**Methods:**

Three hundred and six patients with 373 HCC lesions were enrolled in this study, and underwent biphasic contrast-enhanced scans on a 64-section MDCT. The anatomy of ectopic blood supply, morphological characteristics of HCC including the size, location and pseudocapsule, and history of transcatheter arterial chemoembolization (TACE) therapy were quantitively assessed and statistically analyzed.

**Results:**

Ectopic blood supply was found in 30.8% (115/373) lesions. The ectopic arteries were predominantly composed of inferior phrenic artery (86/115) followed by left and right gastric artery (25/115). Tumor size, location, status of pseudocapsule, and history of TACE therapy could impact the origination of ectopic arteries (all *p*<0.05).

**Conclusion:**

The ectopic feeding arteries of HCC predominantly composed of the perihepatic arteries are associated with the morphological features of the tumor and therapeutic history.

## Introduction

Hepatocellular carcinoma (HCC) is one of the most common malignancies worldwide and is responsible for more than 500,000 deaths every year globally [1]. Radical excision is the ideal method for curing HCC. Unfortunately, the majority of tumors are in the intermediate or advanced stage at the time of diagnosis, leading patients to receive palliative therapy rather than radical excision. Transcatheter arterial chemoembolization (TACE) is an important therapeutic alternative for unresectable HCC [2], [3]. To successfully perform TACE, the feeding arteries of HCC including hepatic artery (HA) and ectopic perihepatic arteries should be evaluated before treatment [4], [5].

Previous studies using digital subtraction angiography (DSA) have found that perihepatic arteries such as internal mammary artery (IMA), intercostals artery (ICA), inferior phrenic artery (IPA), gastric artery (GA), superior mesenteric artery (SMA), gastro duodenal artery (GDA), cystic artery (CA), renal artery (RA), and also lumbar artery (LA) can supply HCC lesions [6]–[12]. Although DSA is considered as the gold standard modality for assessing ectopic blood supply of HCC, its application is limited due to the invasivenese, the cost expense and also the inability to demonstrate all blood vessels in an examination. In recent years, multidetector row computed tomography angiography (MDCTA) has been applied in a wide variety of vascular diseases of the head, thorax, coronary arteries and abdomen [13]–[16]. MDCTA can also be used for displaying hepatic and perihepatic vessels [17]–[21]. In addition, previous findings showed that MDCTA could depict most of the tumor feeding vessels from the intercostal arteries in patients with HCC. MDCTA was also suggested to be conducted for any tumors to locate the feeding vessel before chemoembolization [17], [18]. However, it needs to be confirmed whether the morphological features of HCC and the therapeutic history can affect formation of the ectopic supply.

Thus, we performed this retrospective study to evaluate the anatomy of ecoptic blood supplies of HCC by MDCTA prior to performing TACE, and determine whether the morphological features of HCC and the therapeutic history could affect the occurrence of ectopic feeding arteries.

## Materials and Methods

### Ethics Statement

This consent procedure was approved by the ethics committee of Sichuan Provincial People’s Hospital and West China Hospital of Sichuan University. Obtaining MDCT of 310 patients with HCC was approved by the Institutional Review Board of the Sichuan Provincial People’s Hospital and West China Hospital of Sichuan University. Written informed consent was obtained from each of the 310 patients prior to the study.

### Subjects

From July 2009 to December 2011, 310 consecutive patients with HCC underwent biphasic contrast-enhanced MDCT scans. Of these patients, 4 were abandoned due to the poor image data quality to perform MDCTA and the remaining 306 patients were recruited into our study. The enrolled patients included 197 males and 109 females, and the mean age was 46.3±13.2 years (range from 28–71 years).The time between MDCTA and pathological examination was 1 week to 30 months. The diagnosis were confirmed by pathological examination using fine needle aspiration biopsy in 234 patients, or typical CT features based on the American Association for Study of Liver Diseases (AASLD) guidelines [22] and serum alpha-fetal protein (AFP) in the remaining 72 patients.

### CT Scanning

All patients were scanned on a 64-section MDCT (Philips Brilliance, Philips Medical Systems, Best, and the Netherlands). Before scanning, patients were required to drink 800 to 1,000 ml warm water to completely fill the stomach and the upper abdominal intestine. All patients underwent craniocaudal scanning in the supine position during a single breath hold. The scanning area was from pulmonary hilum level to iliac crest level. A non-contrast upper abdominal scan was performed initially to confirm the location of HCC. Subsequently, a dose of 1.5 ml per kg body weight nonionic contrast material with a concentration of 370 mg of iodine per milliliter (iopamidol 370, Bracco, Milan, Italy) was administered intravenously via the right antecubital vein through a 19-gauge catheter at a rate of 4–5 mL/sec by using an automated injector (Stellant CT Injection Systems, MEDRAD INC, Indianola, USA). A 10 ml saline flush was followed immediately. To perform the optimal arterial phase MDCTA scans, an automatic bolus-triggering software program was systematically applied with a circular region of interest positioned within abdominal aorta above the level of bifurcation of the celiac artery, and the threshold for triggering data acquisition was preset at 100 HU to determine the scanning delay for arterial phase imaging. That is to say, this arterial phase imaging was performed approximately 25–30 sec after the beginning of contrast injection determined by the triggering software program. The scanning was performed with the following parameters: tube voltage of 120 kV, tube current of 220 mAs, field of view (FOV) of 320 mm×320 mm, slice thickness/interval of 5 mm/5 mm, scanning collimator of 0.625 mm, pitch of 1, and matrix of 512×512. The original images were reconstructed with slice thickness of 0.9 mm without interval. Sixty-five sec after the beginning of contrast injection, portal venous phase CT scans were performed to meet the needs of clinical diagnosis. The parameters used for portal venous phase scans were same with those used for arterial phase MDCTA scans.

### Image Analysis

Image data were transmitted to the local workstation (Philips Extended Brilliance workspace workstation, Philips Medical Systems, Best, and the Netherlands) and picture archiving and communication system (PACS) (Siemens AG, Erlangen, Germany) for post-processing and analysis. Thin-slice source images were used to reconstruct perihepatic arteries and hepatic artery (HA) with the techniques including multi-planar reconstruction (MPR), curved planar reconstruction (CPR), maximum intensity projection (MIP) and volume rendering (VR). The reconstructed images were assessed by two experienced abdominal radiologists (the first author possessed a 17-year experience in radiology, and the corresponding author with 24 years of experience in abdominal radiology). The perihepatic arteries included IMA, ICA, IPA, GA, SMA, GDA, CA, RA and LA. Diagnostic criteria of ectopic feeding arteries on MDCTA were as follows: (1) perihepatic arterial branch reached the mass, or (2) ectopic HA could be found although the mass was supplied by HA.

The previous history of TACE therapy was also recorded for further analysis. In addition, we also assessed the morphological characteristics of the primary tumors including the size, location and mass with pseudocapsule or not. For the tumor size, the averaged diameter of the maximal and minimal diameter on the maximal axial slice of the mass was measured on the portal venous phase image. According to the grouping criterion on pathology [23], the lesions were divided into four groups with the averaged diameter set at<3 cm, ≥3 cm and <5 cm, ≥5 cm and <10 cm, and ≥10 cm respectively. According to the anatomic distribution of the primary tumor, the lesions were divided into two groups, i.e., groups with and without liver capsule involved. The criterion for determining the tumor involving liver capsule was the lesion extending beyond the hepatic contour [24]. According to the history of therapy, the lesions were divided into two groups: Non-TACE treated group and TACE treated group. Determining criteria of the tumor undergone TACE therapy included clear history of TACE therapy, and high density of iodized oil deposition in lesions. According to the status of pseudocapsule, lesions were divided into two groups: groups with and without intact pseudocapsule. The criterion for determining lesion with intact pseudocapsule was complete fibrous ring around the lesion, which was isodense or slightly lower density in plain scan and was enhanced in portal-phase enhancement imaging [25].

### Statistical Analysis

Statistical analysis was performed with commercially available statistical software (SPSS, version 15.0 for Windows; SPSS Inc., Chicago, USA). Quantitative variables were presented as mean ± standard deviations. The differences of ectopic arteries among the four groups with different tumor size were evaluated by using a Mann-Whitney rank sum tests, while the differences of ectopic arteries between the two groups were analyzed using a chi-square test. Statistical significance was set at *P<*0.05.

## Results

### The Features of the Hepatic Tumors

In this cohort, 373 lesions were found in 306 patients. All the lesions are grouped according to the tumor size, location, status of pseudocapsule, and the history therapy of the tumor as shown in Table 1.

**Table 1:** Number of tumors in groups.

Groups	Number of lesions (n = 373)
Tumor size (cm)	
<3	34 (9.11)
≥3 and <5	86 (23.06)
≥5 and <10	144 (38.61)
≥10	109 (29.22)
Tumor location	
With liver capsule involved.	199 (53.35)
Without liver capsule involved	174 (46.65)
The status of pseudocapsule	
With intact pseudocapsule	64 (17.16)
Without intact pseudocapsule	309 (82.84)
History of therapy	
With TACE treatment	170 (45.58)
Without TACE treatment	203 (54.42)

Note: Numbers in the bracket are percentages; and TACE = Transcatheter arterial chemoembolization.

### The General Features of The Ectopic Arteries

**Figure 1:**
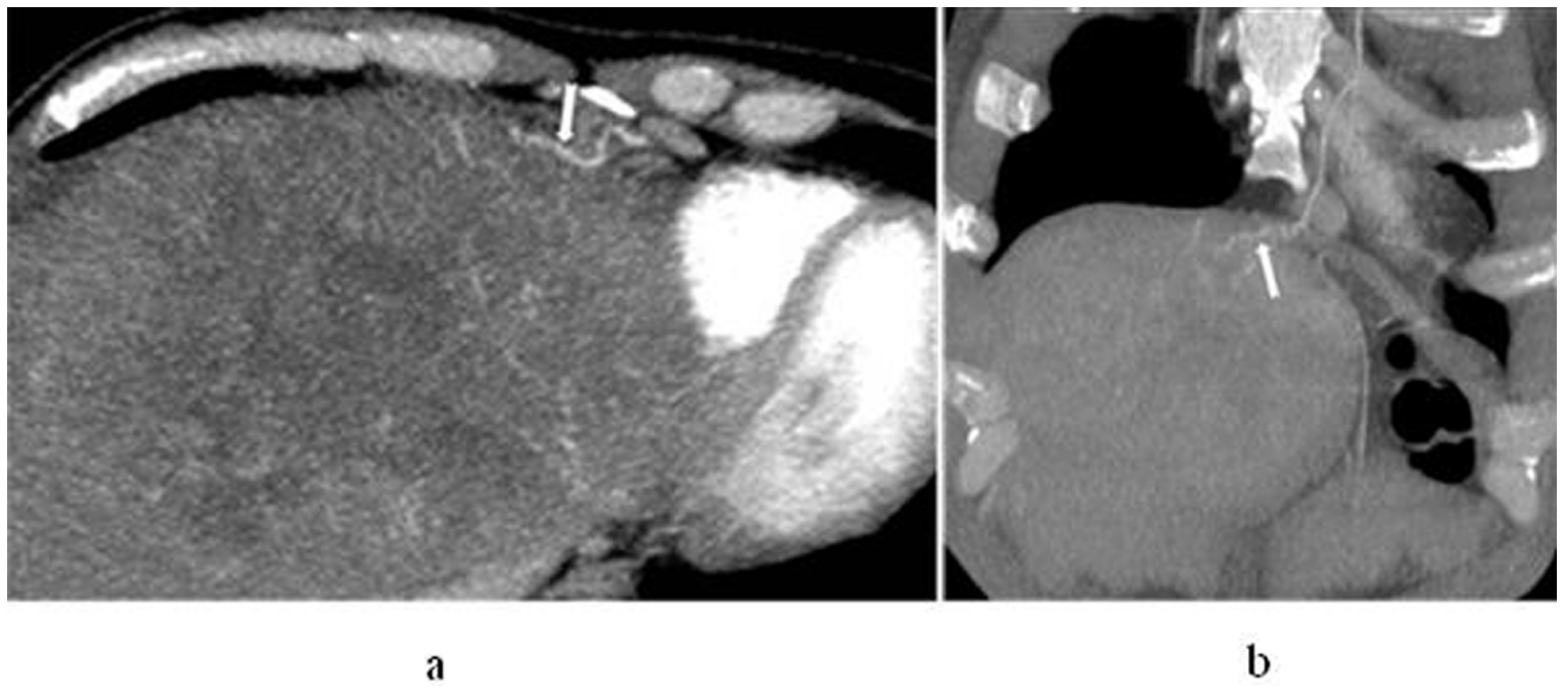
In a 45-year-old man with hepatocellular carcinoma in S8, left internal mammary artery participates in blood supply for the tumour. Axial (a) and coronal (b) maximum intensity projection display that left mammary artery gives off branches into the lesion, and the branches are twist and enlarged (white arrow).

**Figure 2:**
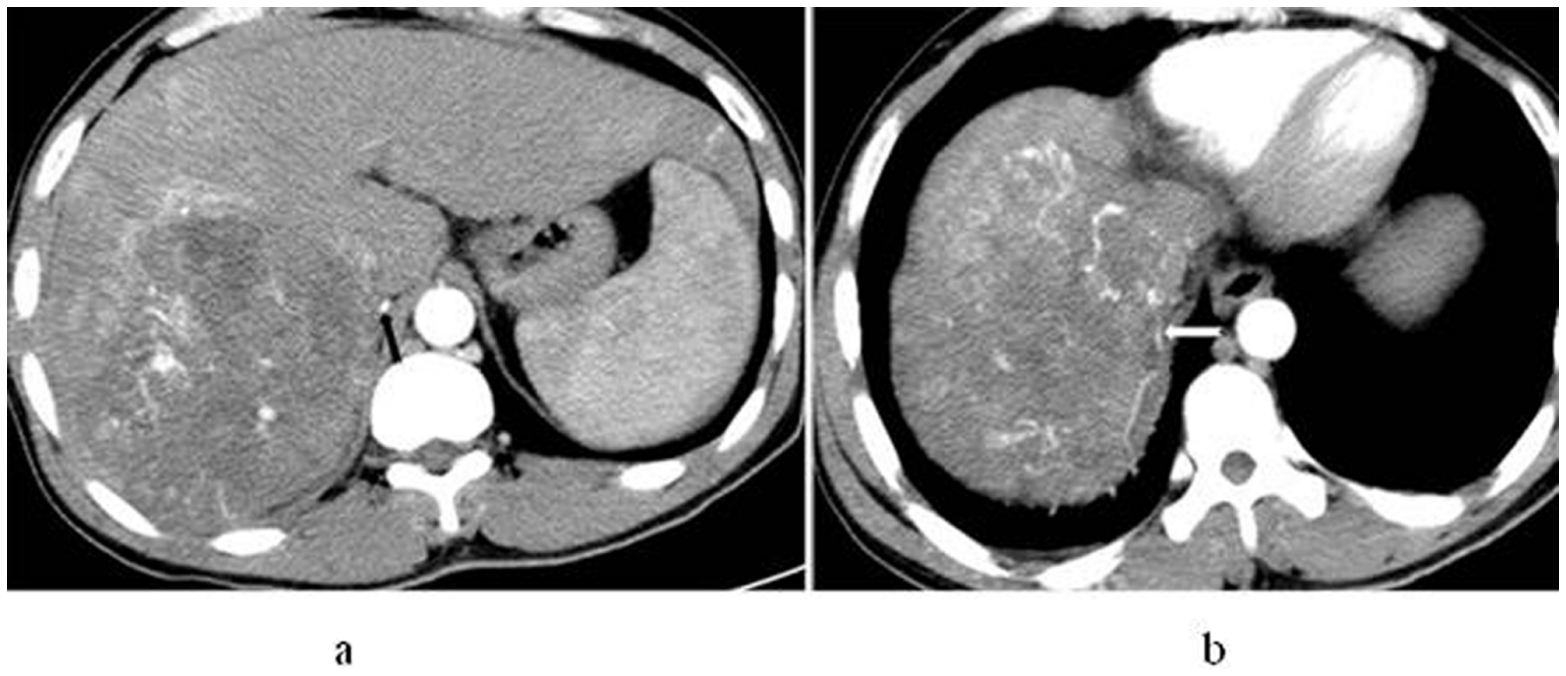
In a 52-year-old man with hepatocellular carcinoma in S7, right inferior phrenic artery participates in HCC blood supply for the tumour. Axial (a) and maximum intensity projection (b) images display the enlarged right inferior phrenic artery (black arrow) and its branches into the lesion (white arrow), respectively.

**Figure 3:**
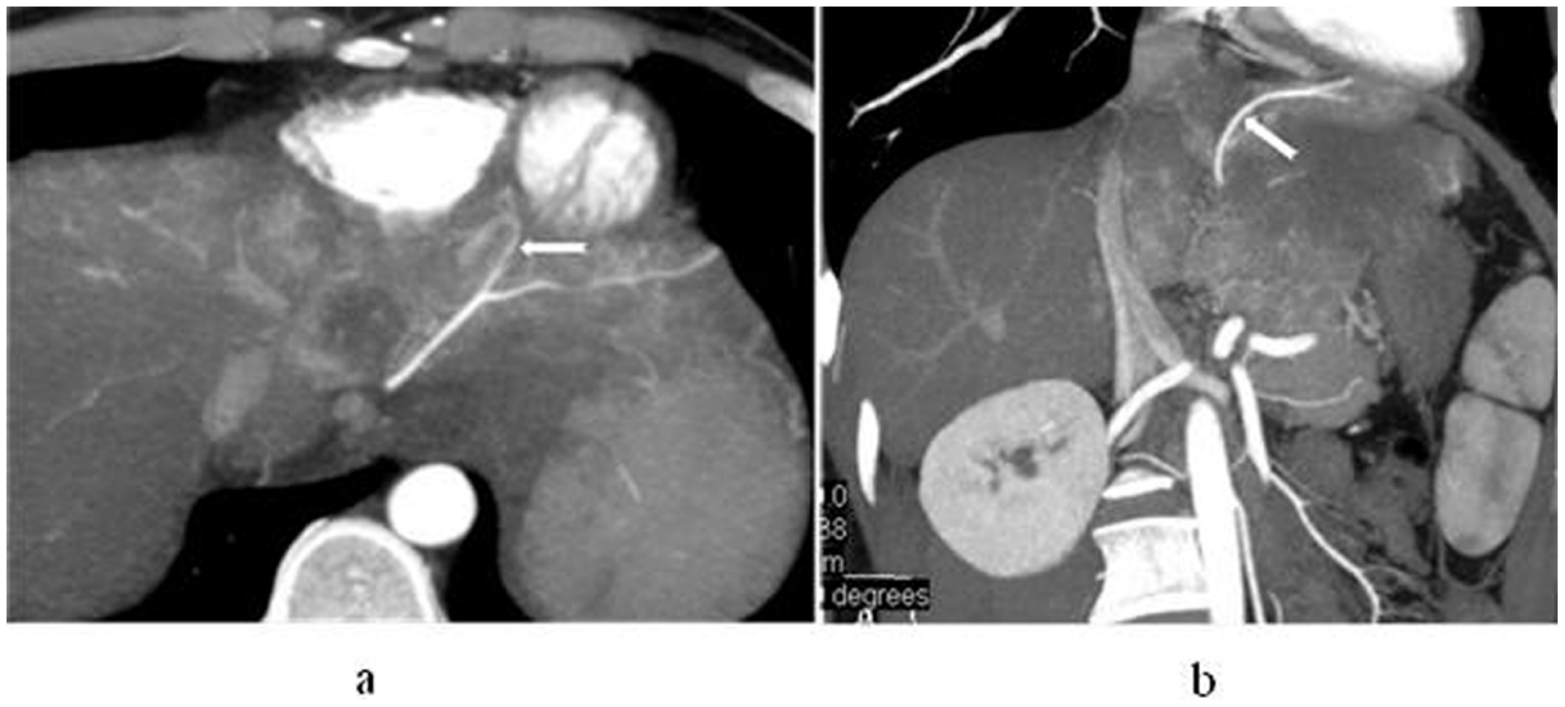
In a 31-year-old man with hepatocellular carcinoma in S2 and S3, left inferior phrenic artery participates in blood supply for the tumour. Axial (a) and coronal (b) maximum intensity projection display that left inferior phrenic artery is enlarged apparently (white arrow) and its branches into the tumor (white arrow).

**Figure 4:**
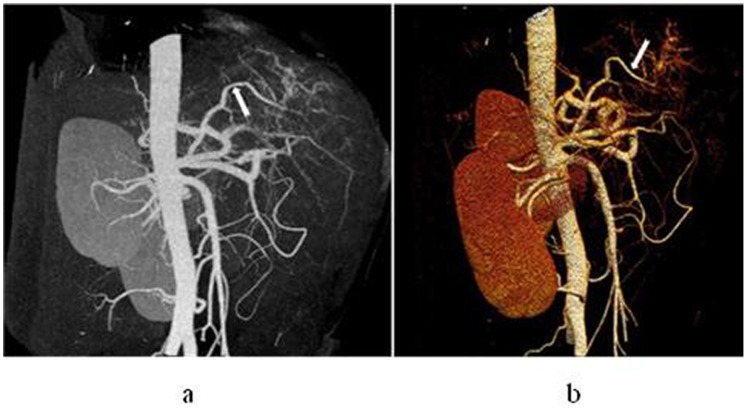
In a 44-year-old man with hepatocellular carcinoma in S2 and S3, left gastric artery participates in blood supply for the carcinoma. Maximum intensity projection (a) and volume rendering technique (b) display that left gastric artery gives off a branch into the lesion and the branch is enlarged apparently (white arrow).

**Figure 5:**
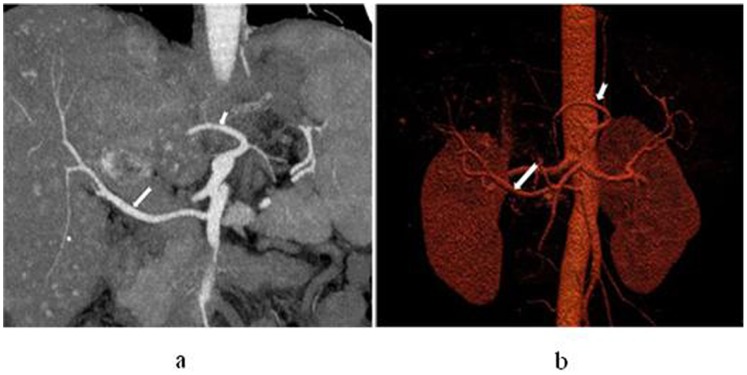
In a 37-year-old woman with hepatocellular carcinoma in right lobe of liver, the variable HA participates in blood supply for the carcinoma. Maximum intensity projection (a) and volume rendering technique (b) display that right HA arise from superior mesenteric artery (long arrow) and left HA arise from left gastric artery (short arrow), which give off branches into the lesion.

**Figure 6:**
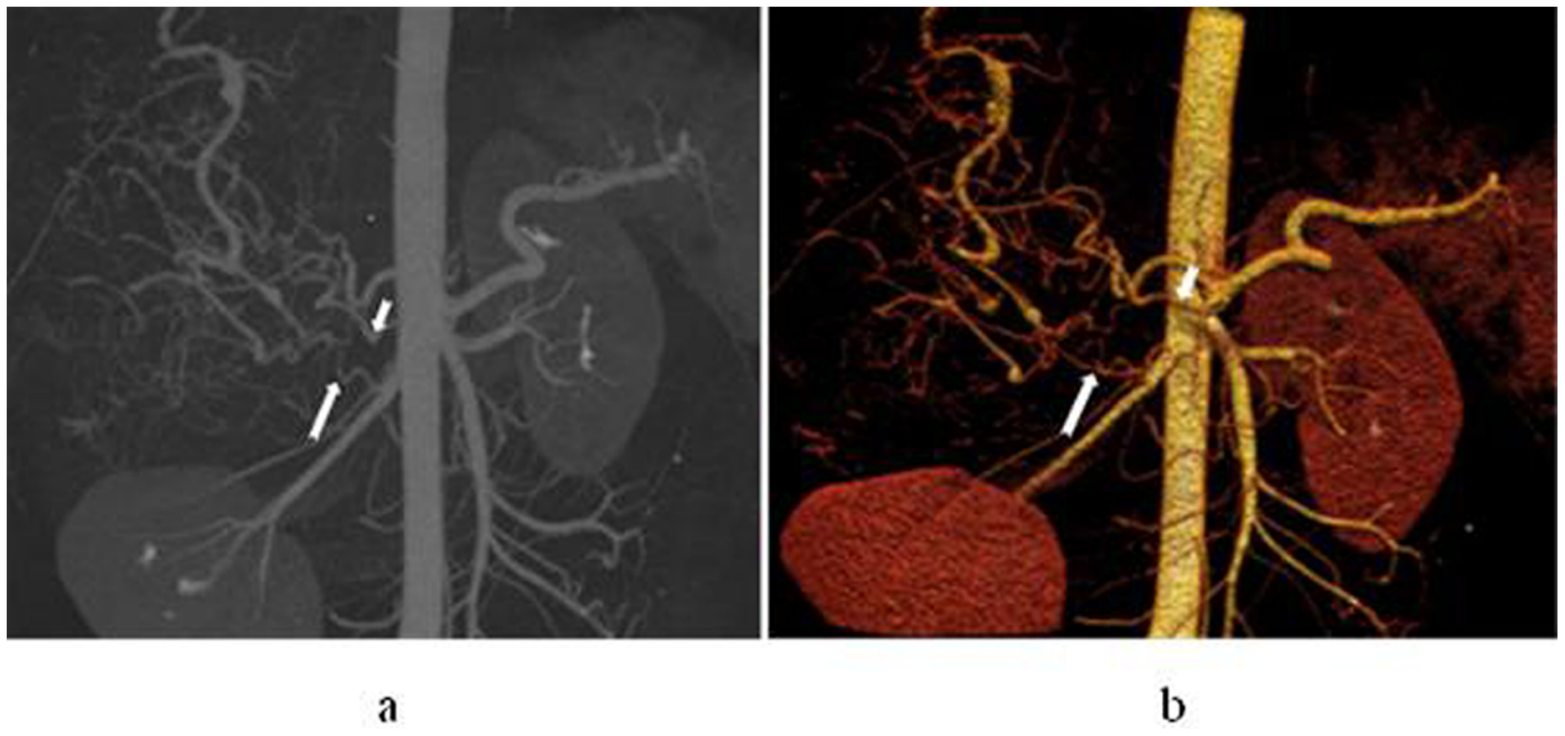
In a 35-year-old man with hepatocellular carcinoma in S6 and S7, right renal artery and adrenal artery participate in blood supply for the carcinoma. Maximum intensity projection (a) and volume rendering technique (b) display that right adrenal artery (short arrow) and the initial segment of right renal artery (long arrow) gives off branches into the tumor.

**Figure 7:**
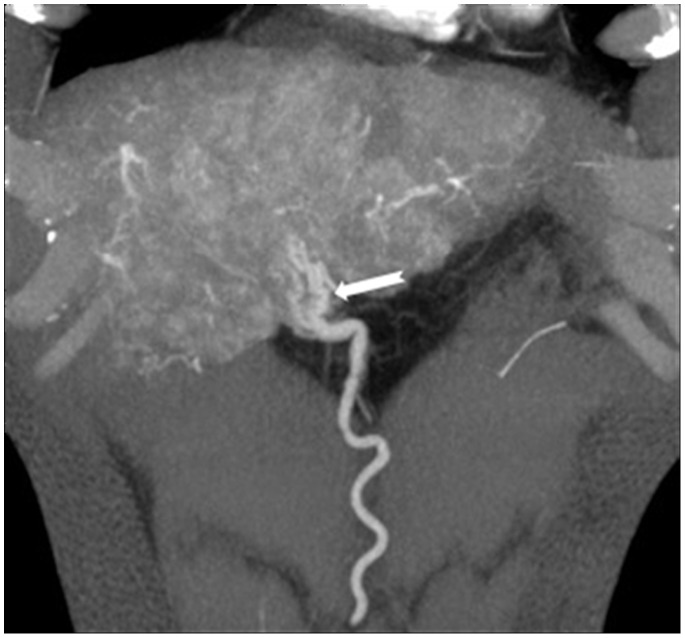
In a 59-year-old man with hepatocellular carcinoma with S5 and S8, abdominal wall artery participates in blood supply for the carcinoma. Maximum intensity projection displays that a large, twist branch of abdominal wall artery enter the tumor (white arrow).

The ectopic arteries of hepatocellular carcinoma are shown in Figure 1–7. In all lesions, 69.2% (258/373) were fed merely by HA, and 30.8% (115/373) were fed by ectopic arteries with or without HA. According to the number of ectopic arteries, 1 ectopic artery was found in 79.1% (91/115) lesions, 2 ectopic arteries were found in 17.4% (20/115) lesions, and 3 ectopic arteries were in 3.5% (4/115). The total number of ectopic arteries feeding the lesions was 143, which are listed in Table 2.

**Table 2:** Number of ectopic blood supply arteries according to their anatomy.

Ectopic blood supply arteries	Number (n = 143)
From lower thorax	
Internal mammary artery	8 (5.59)
Intercostals artery	4 (2.80)
From upper abdomen	
Right inferior phrenic artery	74 (51.75)
Left inferior phrenic artery	12 (8.39)
Left gastric artery	24 (16.78)
Right gastric artery	1 (0.70)
Superior mesenteric artery	1 (0.70)
Gastroduodenal artery	5 (3.50)
Cystic artery	9 (6.29)
Right renal artery	2 (1.40)
Right adrenal artery	1 (0.70)
Right lumbar artery	1 (0.70)
Abdominal wall artery	1 (0.70)

Note: Numbers in the bracket are percentages.

### Ectopic Arteries between Groups

The characteristics of ectopic flood supply in groups with different tumor sizes are presented in Table 3. In general, the larger of the tumor, the more lesions fed by ectopic arteries. Especially, the percentage of ectopic arteries was significantly higher in tumor with size ≥5 cm than that of <5 cm (*P*<0.05). The differences of the number of lesions with ectopic arteries were statistically significant among all the 4 groups with different tumor size (*P*<0.05), except between groups with tumor size <3 cm and with tumor size ≥3 cm and <5 cm (*P*>0.05).

**Table 3:** Ectopic blood supply in tumors according to the tumor sizes.

Tumor sizes	No. of Hepatic tumor (n = 373)	Tumors with ectopic arteries (n = 115)
<3 cm	34 (9.11)	1 (2.94)
≥3 cm and <5 cm	86 (23.06)	5 (5.81)
≥5 cm and <10 cm	144 (38.61)	45 (31.25)
≥10 cm	109 (29.22)	64 (58.72)

Note: Numbers in the bracket are percentages.

The occurrence of ectopic blood supply in group with involved capsule 53.77% (107/199) was significantly higher than that in group without involved capsule 4.60% (8/174) (*P*<0.05). Furthermore, the presence of ectopic blood supply occurred more frequently in group with non-intact pseudocapsule than with intact pseudocapsule (112/309 vs. 3/64, *P*<0.05).

According to the history of TACE therapy, 38.82% (66/170) lesions with history of TACE therapy were found with ectopic arteries, while 24.14% (49/203) lesions without previous TACE therapy were found with ectopic arteries. And the difference was also significant (*P*<0.05).

## Discussion

In the current study, we confirmed that perihepatic arteries participating in HCC blood supply included two parts, i.e., from lower thorax and from upper abdomen. Arteries from lower thorax include IMA and ICA. IMA derives from subclavian artery, and goes down along bilateral-posterior sternum. It gives off anterior intercostal branches; pericardiacophrenic artery, anterior mediastinal artery, pericardial branches, sternal branches and diaphragmatic artery, and ends with epigastria artery. Branches of pericardiacophrenic artery and diaphragmatic artery penetrate diaphragm, and anastomose with branches of IPA and HA which come from upper abdomen [4], [7], [8]. Additionally, lower posterior intercostal artery also anastomoses with IPA. ICA derives from posterior intercostal artery given off by thoracic aorta, and anastomoses with anterior intercostal branches given off by IMA. It has been proved that liver has other blood supply such as IMA and IPA besides HA [26]. Using CT angiography, we found that there were anastomosis between branches of HA and perihepatic arteries.

Arteries from upper abdomen include IPA, left GA (LGA), right GA (RGA), CA, and epiploic branches of GDA, SMA, right RA, right AA, right LA and abdominal wall artery. IPA composed of right and left branches supplies most part of diaphragm within bare area of liver [4], [9]. IPA varies largely, and derives from aortic artery, celiac trunk artery, SMA or RA. Right and left IPA derives from the same or separate trunk [27], [28]. LGA derives from celiac trunk, travels towards the upper left, and runs down along lesser curvature when reaching cardia and anastomoses with RGA which derives from common HA or proper HA. Because the great omentum moves to a large extent, omental branches of GDA given off by common HA may become the ectopic arteries of HCC. SMA, which is relatively far away from the liver, derives from celiac trunk and extends towards lower abdomen. Because of exogenous growth of some HCC, branches close to the liver such as colonic branch may become supplying artery of HCC [12]. Right RA, AA and LA which derive from abdominal aorta locate in retroperitoneal space along with bare area of liver. If HCC invades retroperitoneal space through the bare area of the liver, these three arteries may participate in blood supply of lesion [11].

Furthermore, the anatomic variance of HA is common among people, especially the separate origination of the right and the left HA and the presence of accessory HA [29], [30]. Thus the TACE therapy might be incomplete, unsuccessful and/or even cause complications, if the variant HA wasn’t acknowledged before the therapy. So, it is necessary to evaluate the origination, running and distribution of HA before the therapy. In the past, DSA was the main imaging method for detecting feeding arteries of HCC including HA and ectopic blood supply. However, previous studies reported that MDCT could be used to detect feeding arteries of HCC from IMA and right IPA [8], [10], [31].

In this study, we found that the incidence of HCC supplied by ectopic arteries increased gradually with the increased size of hepatic mass. Wang et al have found that, after repeated chemoembolization, most of the ectopic blood supply in HCC formed among the tumors with the sizes ranging from 5 to 10 cm in diameter [32]. Our findings were consistent with this published report. With the increased size of HCC, HA would be insufficient to supply nutrition for the tumor especially when HA was blocked by intervention radiology. Because of anatomical basis of ectopic blood supply, perihepatic artery would participate in blood supply for HCC.

We also found that the probability of the occurrence of ectopic blood supply for HCC apparently increased when the tumor involved the liver capsule. Liver capsule can prevent the spread of HCC. However, with the growth of the tumor, it would break through the capsule directly or go through the ligament to invade the adjacent organ. HCC is prone to invade retroperitoneal space via bare area of the liver. Once HCC invades adjacent organ, the supplying blood of the involved organ may also supply the HCC lesions by giving off branches or anastomose with the feeding arteries of the lesion. Because peripheral lesions was inclined to involve the liver capsule and grow outward, the probability of ectopic blood supply for peripheral lesions was higher than that of lesion locating in central area of the liver. Nakai et al noted that, regardless of tumor size, when HCCs were located in the ventral hepatic area directly beneath the diaphragm, IMA can serve as a feeding artery in patients with HA occlusion caused by repeated TACE as shown on DSA [7]. If a tumor in central area grew too fast and large, it would also spread toward capsule or even break through the capsule, and invade the adjacent organ, consequently resulting in the occurrence of ectopic blood supply.

To our knowledge, there were no reports about the effect of tumor growth pattern on the occurrence of ectopic blood supply. In this study, we found that the incidence of the ectopic blood supply without intact pseudocapsule was higher than those with pseudocapsule. Pseudocapsule which is the fibrous tissue membrane enveloping the tumor has some blocking effects on the spread of tumor and ectopic blood supply entering into the mass. If pseudocapsule is not intact, or the mass has no pseudocapsule, it is easy for the tumor to invade adjacent structure or the ectopic blood supply to enter the tumor [33]. It should be noted that we also found no ectopic blood supply in lesions with intact pseudocapsule even when its size was more than 10-cm diameter and located at the edge area of the liver.

TACE has been widely used in the management of hepatocellular carcinoma because of its reliable effectiveness on this tumor since 1983 [34]. TACE played its role by blocking blood supply and injecting drugs into feeding artery of the tumor or into the lesion. After HA was blocked, extra hepatic arteries may have opportunities to participate in blood supply of the tumor [35]. Because HCC is characterized by synchronous and asynchronous multicenter originations, which might cause the tumor to relapse after TACE therapy, the blood supply from the artery was replaced by that from perihepatic arteries due to the asynchronous origination and recurrent lesions when HA were blocked.

Our study has some limitations. First, the DSA, the golden standard for vessels, were not available for the patients due to practical reason. However, MDCT had been proved to be accurate in detecting feeding arteries of HCC [31]. Second, the number of patients who received TACE was small, though we showed the potential impact of this therapy on ectopic blood supply of HCC. Further studies should be conducted in supporting this finding. Third, although the diagnosis of some HCC lesions did not depend on pathology, they could be confirmed by typical medical history, AFP indicator and imaging feature. All patients recruited in this study had increased AFP, and typical CT feature, so the chances of misdiagnose were very little.

In conclusion, MDCTA is able to provide comprehensive characteristics of the ectopic blood supply of HCC and its related risk factors. The formation and patterns of the ectopic blood supply for HCC are closely related to tumor size, superficially anatomic location of tumors, status of pseudocapsule and multiple chemoenbolization. These findings would be helpful for the pre-TACE preparation for patients with HCC.
